# Recognition-Mediated Assembly of Quantum Dot Polymer Conjugates with Controlled Morphology

**DOI:** 10.3390/ijms12096357

**Published:** 2011-09-23

**Authors:** Vikas Nandwana, Chandramouleeswaran Subramani, Serkan Eymur, Yi-Cheun Yeh, Gulen Yesilbag Tonga, Murat Tonga, Youngdo Jeong, Boqian Yang, Michael D. Barnes, Graeme Cooke, Vincent M. Rotello

**Affiliations:** 1 Department of Chemistry, University of Massachusetts Amherst, 710 North Pleasant Street, Amherst, MA 01003, USA; E-Mails: nandwana@chem.umass.edu (V.N.); csubrama@chem.umass.edu (C.S.); yyeh@chem.umass.edu (Y.-C.Y.); gyesilba@chem.umass.edu (G.Y.T.); mtonga@chem.umass.edu (M.T.); jeong@chem.umass.edu (Y.J.); mdbarnes@chem.umass.edu (M.D.B.); 2 Department of Chemistry, Middle East Technical University, Ankara 06531, Turkey; E-Mail: eymur@chem.umass.edu; 3 Department of Physics, University of Massachusetts Amherst, 710 North Pleasant Street, Amherst, MA 01003, USA; E-Mail: boqian@physics.umass.edu; 4 Glasgow Centre of Physical Organic Chemistry, WestCHEM, School of Chemistry, University of Glasgow, Joseph Black Building, Glasgow G12 8QQ, UK; E-Mail: Graeme.Cooke@glasgow.ac.uk

**Keywords:** molecular recognition, nanoparticles, hydrogen bonding, self-assembly, nanocomposites, quantum dots

## Abstract

We have demonstrated a polymer mediated “bricks and mortar” method for the self-assembly of quantum dots (QDs). This strategy allows QDs to self-assemble into structured aggregates using complementary three-point hydrogen bonding. The resulting nanocomposites have distinct morphologies and inter-particle distances based on the ratio between QDs and polymer. Time resolved photoluminescence measurements showed that the optical properties of the QDs were retained after self-assembly.

## 1. Introduction

Quantum dots (QDs) have received extensive attention in recent years owing to their size-dependent optical and electrical properties [1–7]. In particular, these nanocrystals show potential applications in optoelectronic devices such as light-emitting diodes [4,5], photodetectors [6–8], field-effect transistors [9], photovoltaic cells [10,11] and photorefractives [12]. To utilize the QDs for these device fabrications, they should be assembled in morphologically controlled and highly ordered structures [13–18]. These highly ordered structures have been prepared using polymer matrices through the self-assembly of QDs *via* a variety of host-guest interactions [19–23]. However, the control of QD assembly still remains a major challenge due to the indiscriminate aggregation and phase separation, which results in a decay of QD photoluminescence [24].

**Scheme 1:**
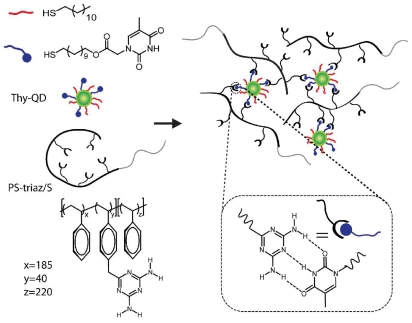
Schematic illustration of the assembly process between Thy-QD and PS-triaz/S *via* three-point hydrogen bonding.

Our group has explored the use of polymer mediated “bricks and mortar” strategy to control the nanoparticle self-assembly using non-covalent interactions [25,26]. In this strategy, nanoparticles functionalized with recognition elements serve as the bricks, while polymers bearing complementary functionality serve as the mortar. The irregularities in the size and shape of the structured aggregates are compensated by the conformational flexibility of the polymer, permitting the efficient propagation of order in the self-assembly process. In previous studies using gold and magnetic nanoparticles, we have shown that the polymer mediated self-assembly of nanoparticles provides structures with increased ordering and controlled inter-particle spacing [27–33]. Here, we report the polymer mediated self-assembly of QDs using complementary three-point hydrogen bonding. We utilized diaminotriazine-functionalized diblock copolymer (PS-triaz/S) and thymine functionalized QDs (Thy-QD) to generate self-assembled nanostructures (Scheme 1). This strategy also provided controlled morphologies and tunable inter-particle distances for the self-assembled aggregates. Time resolved photoluminescence measurements show that the optical properties of the QDs were retained after self-assembly.

## 2. Results and Discussion

The self-assembly was driven by the well-established diaminotriazine-thymine three-point hydrogen bonding complementary interactions [30–33]. Addition of PS-triaz/S to Thy-QD in non-competitive solvents such as dichloromethane and chloroform resulted in the formation of a precipitate. PS-triaz/S folds into a highly compact structure due to intramolecular hydrogen bonds between the triazines in non-polar solvents [34]. Multivalent interactions of PS-triaz/S with Thy-QD induce the unfolding of the compact structure of the polymer, exposing further triazine recognition units. This allows PS-triaz/S to interact with further Thy-QD units, propagating the assembly process (Scheme 1). The experiment was comprised of three samples with Thy-QD:PS-triaz/S molar ratios (based on recognition units) of 10, 1 and 0.1.

**Figure 1:**
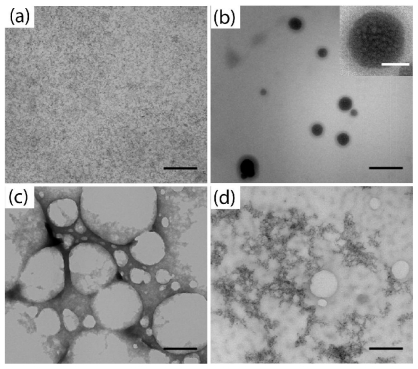
TEM of (**a**) Thy-QD and Thy-QD/PS-triaz/S conjugates with QD to polymer ratio; (**b**) 10:1; (**c**) 1:1; and (**d**) 1:10. Scale bar 200 nm (inset Figure 1(b) 50 nm).

Transmission electron microscopy (TEM) analysis of these precipitates revealed that varying the ratios of Thy-QD to PS-triaz/S resulted in distinct morphologies (Figure 1). Higher ratios of Thy-QD to PS-triaz/S (10:1) resulted in densely packed spherical aggregates ranging from 50 to 200 nm in diameter (Figure 1(b)) while lower ratios (1:10) led to loosely networked aggregates embedded in a polymer matrix (Figure 1(d)). An equimolar ratio of Thy-QD to PS-triaz/S resulted in dense web like structures (Figure 1(c)). Along with morphologies, packing of QDs in the assemblies could be readily discerned at different QD to polymer ratios.

**Figure 2:**
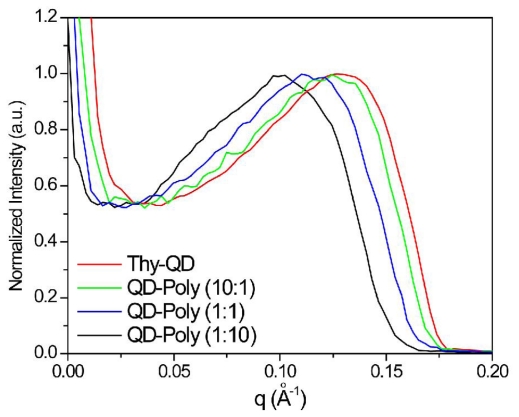
SAXS plots of Thy-QD (red) and Thy-QD/PS-triaz/S conjugates with QD to polymer ratio 10:1 (green), 1:1 (blue), and 1:10 (black).

Quantification of inter-particle distance in the QD aggregates was obtained using small angle X-ray scattering (SAXS) (Figure 2). Thy-QD drop cast from dichloromethane had a scattering vector (q) with maximum intensity at 0.131 Å^−1^. This *q* value was converted to inter-particle distance (d) through the relationship (q = 2π/d) with the resultant value of 4.79 nm. This result is concurrent with 3.5 nm QDs spaced only by the monolayer of ligands. When PS-triaz/S was mixed to assemble Thy-QD, the scattering vector (q) peak value decreased as PS-triaz/S concentration was increased in the assemblies, confirming the increase in inter-particle distance (Table 1). The SAXS data of QD aggregates suggests that QDs were regularly dispersed within the polymer matrix and provides evidence that individual polymer chains weaved the QDs together as shown in assembly formation hypothesis in Scheme 1.

**Figure 3:**
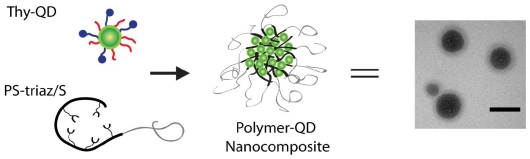
Schematic illustration of corona formation outside spherical QD aggregates. Scale bar is 100 nm.

Spherical QD aggregates formed using this approach are truly multi-scale constructs, structured on the molecular and nanometer scale (Figure 1(b)). The light contrast outside the spherical aggregates shows that the interacting part of the diblock co-polymer aggregates the QDs at the core whereas the non-interacting part forms the corona (Figure 3). Hence, it is also possible to control the size of the spherical aggregates by changing the length of interacting part of the block copolymer [31].

**Figure 4:**
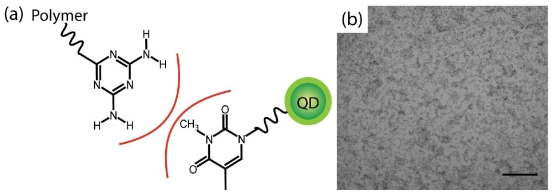
(**a**) Schematic of non-hydrogen-bonding control of MeThy-QD which fails to bind with the PS-triaz/S; (**b**) TEM of MeThy-QD and PS-triaz/S mixture, shows no aggregation. Scale bar is 200 nm.

To provide a control system that cannot participate in hydrogen bonding, N(3)-methyl thymine functionalized QDs (MeThy-QD) were prepared (Figure 4(a)). In contrast to Thy-QD, no precipitation was observed upon addition of PS-triaz/S to the MeThy-QD (Figure 4(b)). The lack of aggregation observed in the control system demonstrates that the precipitation observed upon the addition of PS-triaz/S to Thy-QD was the result of specific hydrogen-bond interactions between the thymine and the diaminotriazine.

**Figure 5:**
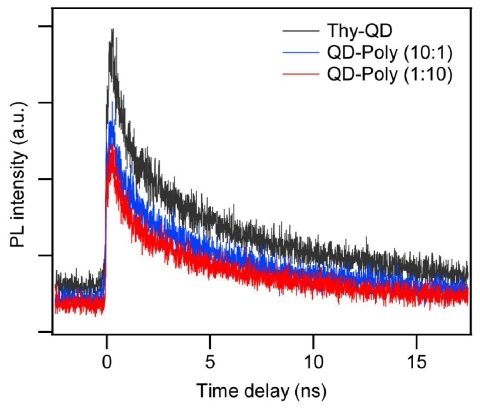
PL decay dynamics of Thy-QD (black) and Thy-QD/PS-triaz/S conjugates with QD to polymer ratio of 10:1 (blue) and 1:10 (red).

Figure 5 shows time-resolved photoluminescence (PL) decay curves of Thy-QD and its aggregates with Thy-QD:PS-triaz/S at a ratio of 10 and 0.1. PL decay dynamics for all the samples were taken at the peak emission wavelength (537 nm) with a bandwidth of 5 nm. After an initial fast decay, the PL dynamics showed a slow decay, which are typical for measurements of QDs ensembles [35,36]. By fitting the decay curves as a biexponential decay with the function A_1_e^−1/τ1^ + A_2_e^−1/τ2^, we obtained the lifetimes for fast (τ1) and slow (τ2) decay components as shown in the Table 2 where A_1_ and A_2_ are their relative proportions. The QDs, after assembly, showed a negligible change in PL decay curves. The negligible change in the lifetime of excitons in QD polymer aggregates indicates no significant change in optical properties of the QDs.

## 3. Experimental Section

### Synthesis of TOPO functionalized CdSe/ZnS quantum dots (QDs)

CdSe/ZnS core-shell QDs of diameter 3.5 nm were prepared according to the reported procedure [37,38]. Briefly, CdO (0.0514 g, 0.4 mmol), tetradecyl phosphonic acid (TDPA) (0.2232 g, 0.8 mmol) and trioctylphosphine oxide (TOPO) (3.7768 g, 9.77 mmol) were loaded into a 50 mL three-neck flask and heated to 350 °C under an argon (Ar) flow. After 3 h, the temperature was decreased to 270 °C and the Se solution (Se (0.042 g, 0.53 mmol) in 2.4 mL trioctyl phosphine (TOP)) was swiftly injected into the hot solution. The CdSe QDs were purified and precipitated with chloroform (CHCl_3_) and methanol, and finally dissolved in CHCl_3_. Then, the CdSe core solution was mixed with TOPO (4 g, 10.3 mmol) and hexadecylamine (HDA) (1.5 g, 6.2 mmol) and heated to 150 °C for 1 h. Diethylzinc (ZnEt_2_) (1.6 mL, 1.6 mmol) in 2.4 mL TOP and hexamethyl-disilathiane (TMS)_2_S (0.278 mL, 1.3 mmol) in 5.25 mL TOP were used as a shell solution. After injecting the shell solution, the QD mixture was reacted for 1 h at 100 °C. The resulting CdSe/ZnS QDs were purified and precipitated with CHCl_3_ and methanol, and finally stored in toluene.

### Ligand exchange reaction

TOPO functionalized QDs (30 mg) were mixed with HS-(CH_2_)_5_-TEG ligands (90 mg) in a dichloromethane (DCM) solution (10 mL) and stirred for 24 h under Ar gas at room temperature. QDs were precipitated in hexanes and redissolved in 10 mL methanol. Thymine functionalized alkanethiol (42 mg) and dodecanethiol (200 μL) were added to the QD solution. The reaction mixture was purged with Ar gas and stirred for 2 days at 39 °C in a closed vial. Then, the solvent from the mixture was evaporated and QDs were dispersed in DCM. After precipitation with methanol and centrifugation, thymine functionalized QDs were redispersed in DCM. NMR showed 95% dodecanethiol and 5% thymine-functionalized alkanethiol present on the particles (see the ESI). N(3)-methylthymine-functionalized QDs were made using the same procedure.

### Synthesis of diblock co-polymer [32]

Preparation of **Poly**((styrene-***ran***-*p*-chloromethyl styrene)-***b***-styrene).

A mixture of the (2,2,6,6-tetramethylpiperidin-1-yl) oxidanyl (TEMPO) (61.1mg, 0.39 mmol), styrene (4.0 g, 38.4 mmol) and *para*-chloromethyl styrene (5.8 g, 38 mmol) were degassed by three freeze/thaw cycles, sealed under argon, and heated at 125 °C under nitrogen for 7 h. The solidified reaction mixture was then dissolved in dichloromethane (10 mL) and precipitated into methanol (300 mL). The precipitate was then collected by vacuum filtration and dried to give the desired **Poly**(styrene-***ran***-*p*-chloromethyl styrene), as a white solid (3.8 g, 72% yield), Mn = 22902, PD = 1.42.

The **Poly**(styrene-***ran***-*p*-chloromethyl styrene) as starting block (1 g, 0.043 mmol) was then redissolved in styrene (10 g, 96 mmol) and the polymerization reaction mixture was heated at 130 °C for 1 h. The solidified reaction mixture was then dissolved in dichloromethane (10 mL) and precipitated into methanol (300 mL). The precipitate was then collected by vacuum filtration and dried to give the desired block copolymer, as a white solid (1.70 g, 89% yield), Mn = 43254, PD = 1.93.

A solution of **Poly**((styrene-***ran***-*p*-chloromethyl styrene)-***b***-styrene) (1.70 g, 0.039 mmol), sodium cyanide (0.61 g, 1.23 mmol), and DMF (10 mL) was heated at 70 °C for 48 h under Ar gas. The resulting heterogeneous mixture was filtered and the filtrate concentrated under reduced pressure. The concentrated solution was precipitated into water. The solid product (1.41 g, 83%) was collected by filtration, washed with water and CH_2_Cl_2_, and dried under vacuum.

**Poly**((styrene-***ran***-*p*-methyldiaminotriazine styrene)-***b***-styrene)

A solution of polymer (0.75 g, 0.17 mmol), dicyandimide (0.55 g, 6.5 mmol), and KOH (0.081 g, 1.46 mmol) in 2-propanol (5 mL) was refluxed for 20 h. A precipitate formed as the reaction proceeded. After evaporating 2-propanol under reduced pressure, the resulting crude product was stirred in boiling water for 30 min. The solution was cooled to room temperature and then filtered to collect a cream-colored solid (0.638 g, 85%).

### Preparation of QD-polymer aggregates

1 μM solution QDs was added in 0.1, 1 and 10 μM PS-triaz/S solutions. The concentration was calculated based on the recognition units. The volume of both the polymer and QDs were kept the same (1 mL) in each mixture. After 2 h, the precipitate was separated by centrifugation and redispersed in dichloromethane.

### Characterization

TEM images were acquired on a JEOL 7C operating at 80 keV. All the polymers and ligands were characterized by NMR (see the ESI). NMR spectra were recorded at 400 MHz on a Bruker AVANCE 400 instrument. For SAXS, Cu Kα X-rays (1.54 Å) were generated in an Osmic MaxFlux source with a confocal multilayer optic (OSMIC, Inc.). Images were taken with a Molecular Metrology, Inc., camera consisting of a three pinhole collimation system, 150 cm sample-to-detector distance (calibrated using silver behenate), and a two-dimensional, multiwire proportional detector (Molecular Metrology, Inc., Northampton, MA, USA). Two-dimensional images were reduced to the one dimensional form using angular integration. The scattering intensity is presented as a function of the wave vector q = (4π/λ) sin(2θ/2), where 2θ is the scattering angle, and λ is the Cu Kα radiation wavelength. Particle spacing (*d*) was calculated from Gaussian fits to the principal scattering maxima of the Lorentz-corrected intensities using d = 2π/q. For time resolved PL measurements, Thy-QD and its aggregates were dropcasted on glass substrate. The samples were excited using 407 nm pulsed diode laser with a repetition rate of 40 MHz and a pulse width of 50 ps. The collected sample PL was spectrally dispersed with a monochromator, detected with an avalanche photodiode (APD, id Quantique id100-50) for photon counting, and analyzed with a time-correlated single photon counting (TCSPC) system (PicoQuant PicoHarp 300). The laser and detector systems provided a 70 ps time resolution in time-resolved PL measurements.

## 4. Conclusions

In summary, we have demonstrated polymer mediated assembly of QDs using a “bricks and mortar” strategy to create structured aggregates of QDs. This process is based on three-point hydrogen bonding between diaminotriazine functionalized diblock co-polymers and thymine functionalized QDs. The morphology as well as inter-particle distance of these aggregates was controlled by tuning molar ratios between the QDs and the polymer while retaining the optical properties of the QDs. Also, the ease of modification of both the nanoparticle and polymer components makes this methodology well suited for the creation of diverse combinations of QD–polymer nanocomposite which might be useful in optoelectronic applications.

## Figures and Tables

**Table 1:** Peak scattering factor (q) and inter-particle distance (d) values for Thy-QD/PS-triaz/S conjugates with QD to polymer ratio 10:1, 1:1, and 1:10.

	Thy-QD	Thy-Poly (10:1)	Thy-Poly (1:1)	Thy-Poly (1:10)
q Å^−1^	0.131	0.126	0.115	0.099
d Å^−1^	4.792	4.955	5.425	6.333

**Table 2:** Fit parameters for decay curves shown in Figure 5.

	A_1_	τ1 (ns)	A_2_	τ2 (ns)
Thy-QD	0.45	0.61	0.55	7.0
Thy-Poly (10:1)	0.43	0.60	0.57	6.40
Thy-Poly (1:10)	0.45	0.60	0.55	6.56
